# Closed Loop Obstruction from Epiploic Appendage Adhesion Mimicking Pericecal Internal Hernia

**DOI:** 10.1155/2018/4767516

**Published:** 2018-09-23

**Authors:** Fatima Sharif, Paul Samuel Sander, Ali Sharif, Grace Montenegro, Robert Garrett

**Affiliations:** ^1^University of Missouri Kansas City School of Medicine, 2411 Holmes Street, Kansas City, Missouri 64108, USA; ^2^Saint Louis University School of Medicine, 3635 Vista Blvd., St. Louis, MO 63110, USA

## Abstract

Internal hernias involve herniation of viscera into an abdominal compartment through a defect in the mesentery or peritoneum. Herniation may occur through normal anatomic structures or through pathologic defects secondary to congenital abnormality, inflammation, trauma, or surgery. Patients with an internal hernia most commonly present with acute bowel obstruction. While internal hernia is an uncommon cause of bowel obstruction, making up approximately 0.2-0.9% of cases (Choi, 2017), the incidence is increasing due to greater use of techniques such as Roux-en-Y for liver transplant and gastric bypass. There are multiple types of internal hernia, including paraduodenal, Foramen of Winslow, sigmoid mesocolon, pericecal, transmesenteric, transomental, supravesical, and pelvic. We present a case in which a transverse colon epiploic appendage adhesion to the ascending colon mesentery resulted in a closed loop obstruction mimicking a pericecal internal hernia. Radiologists should be aware of the imaging findings of closed loop obstruction related to internal hernia and maintain a high index of suspicion in patients with history of prior abdominal surgery presenting with bowel obstruction. It is useful for radiologists to understand that adhesions may result in internal hernias, which mimic the classically described categories.

## 1. Introduction

Internal hernias involve herniation of viscera into an abdominal compartment through a defect in the mesentery or peritoneum. Herniation may occur through normal anatomic structures or through pathologic defects secondary to congenital abnormality, inflammation, trauma, or surgery. Patients with an internal hernia most commonly present with acute bowel obstruction. While internal hernia is an uncommon cause of bowel obstruction, making up approximately 0.2-0.9% of cases [1], the incidence is increasing due to greater use of techniques such as Roux-en-Y for liver transplant and gastric bypass.

There are multiple types of internal hernia, including paraduodenal, Foramen of Winslow, sigmoid mesocolon, pericecal, transmesenteric, transomental, supravesical, and pelvic. We present a case in which a transverse colon epiploic appendage adhesion to the ascending colon mesentery resulted in a closed loop obstruction mimicking a pericecal internal hernia.

## 2. Case Presentation

**Figure 1:**
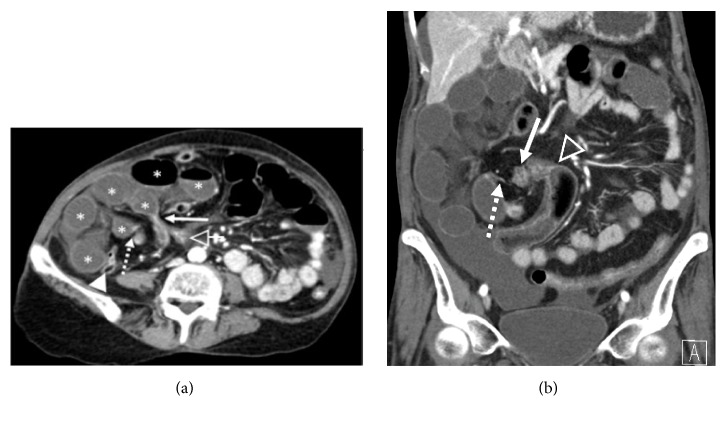
Contrast enhanced CT of the abdomen and pelvis demonstrating closed loop obstruction in the right lower quadrant. (a) Axial CECT demonstrating a proximal transition point within the proximal ileum (dashed arrow) and a nearby distal transition point within the distal ileum (solid arrow). The dilated bowel loops (*∗*) are anterolateral to the cecum (open arrowhead) and anterior to the ascending colon (closed arrowhead). (b) Coronal CECT image demonstrating multiple dilated small bowel loops confined to the right abdomen. Note the diminished enhancement of the dilated small bowel loops.

**Figure 2:**
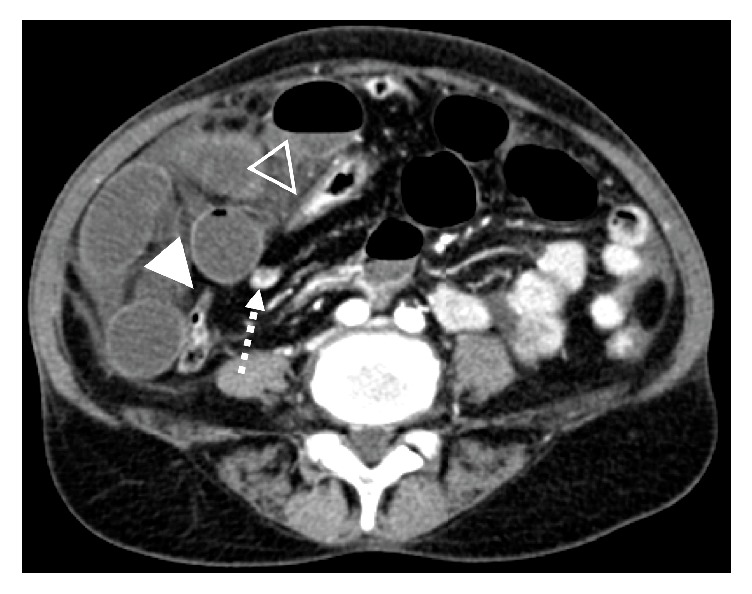
Axial CECT image demonstrating mucosal hyperenhancement of the ileum at the proximal transition point related to ischemia or decompressed state (dashed arrow). Kinking of the ascending colon (closed arrowhead) and transverse colon (open arrowhead) was retrospectively felt to represent the site of the epiploic appendage adhesion.

**Figure 3:**
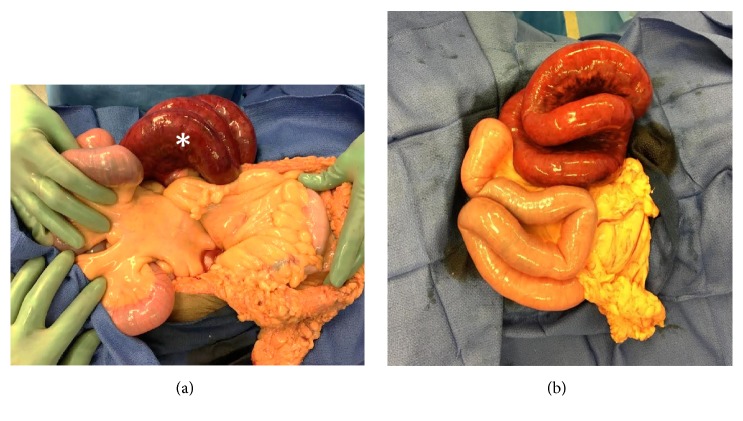
Intraoperative images. (a) and (b) Closed loop obstruction was detected intraoperatively with nonviable bowel (*∗*) which was subsequently resected.

A 58-year-old female presented to the emergency department with intermittent, crampy right-sided abdominal pain, nausea, and vomiting, which began approximately 18 hours previously. Her past medical history was significant for hypertension and her surgical history included a thyroidectomy for treatment of thyroid cancer and a Caesarean section. A contrast enhanced CT abdomen and pelvis was obtained, demonstrating multiple fluid-filled, dilated small bowel loops in the right abdomen, which were predominantly anterolateral to the ascending colon and cecum (Figure 1). In addition, two transition points were identified in the right lower quadrant, with one transition point at the distal ileum just proximal to the cecum and a second transition point in the proximal ileum. The two transition points were in close proximity to each other, indicative of closed loop obstruction. Decreased wall enhancement of the dilated small bowel loops was concerning for ischemia. Mucosal hyperenhancement of the ileum at the proximal transition point was felt to relate to ischemia or decompressed state (Figure 2). Given the patient's symptoms and findings of closed loop obstruction on CT, the patient was taken to operating room. In the operating room, an internal hernia with closed loop obstruction was confirmed and resulted from herniation of small bowel through an adhesion of a transverse colon epiploic appendage to the ascending colon mesentery. The herniated small bowel was nonviable and a total of 60 cm of small bowel was resected (Figure 3). Retrospectively, kinking of the ascending and transverse colon could be seen on the initial abdominal CT and was felt to correspond with the site of adhesion (Figure 2).

After resection, the patient's small bowel was left in discontinuity and an abdominal wound-vac was placed. The following day, the patient returned to the operating room, at which time the terminal ileum was also found to be nonviable. An ileocecectomy with enterocolonic anastomosis was performed.

The patient had a complicated postoperative course, but was ultimately discharged approximately two weeks after the initial surgery.

## 3. Discussion

Based on the imaging findings in this case, this internal hernia was thought to represent a pericecal hernia. Pericecal hernias make up approximately 0.1-6.6% of internal hernias [2]. There are four pericecal recesses that have the potential to become hernia orifices: inferior ileocecal, superior ileocecal, paracolic, and retrocecal, with retrocecal being the most common location of herniation. These hernias occur when loops of ileum herniate through one of these orifices and extend into the right paracolic gutter. Clinical diagnosis may prove difficult, especially in chronic incarceration when symptoms can be confused with inflammatory bowel disease, appendiceal disorders, or other causes of small bowel obstruction [3]. Like most hernias, severity of symptoms relates to duration, reducibility, and presence or absence of incarceration [4].

Clinical manifestations include paroxysmal epigastric discomfort, abdominal distention, colicky periumbilical pain, and nausea and vomiting with meals [4]. To diagnose these hernias, abdominal radiographs can initially be helpful, especially to evaluate for the presence of bowel obstruction [5]. Computed tomography is most often used to definitively diagnose internal hernia. A common CT appearance of pericecal internal hernia is a cluster of fluid-filled, dilated loops of small bowel lateral to the cecum and anterior to the ascending colon, as in this case. While this case mimicked a pericecal internal hernia, operative findings were consistent with herniation of small bowel through an adhesion of a transverse colon epiploic appendage to ascending colon mesentery. Radiologists should be aware of the imaging findings of closed loop obstruction related to internal hernia and maintain a high index of suspicion in patients with history of prior abdominal surgery presenting with bowel obstruction. It is useful for radiologists to understand that adhesions may result in internal hernias, which mimic the classically described categories.
